# Low Density Nanocellular Polymers Based on PMMA Produced by Gas Dissolution Foaming: Fabrication and Cellular Structure Characterization

**DOI:** 10.3390/polym8070265

**Published:** 2016-07-18

**Authors:** Judith Martín-de León, Victoria Bernardo, Miguel Ángel Rodríguez-Pérez

**Affiliations:** Cellular Laboratory (CellMat), Universidad de Valladolid, Valladolid 47011, Spain; vbernardo@fmc.uva.es (V.B.); marrod@fmc.uva.es (M.Á.R.-P.)

**Keywords:** nanocellular polymer, nanocellular foam, gas dissolution foaming, confinement, PMMA

## Abstract

This paper describes the processing conditions needed to produce low density nanocellular polymers based on polymethylmethacrylate (PMMA) with relative densities between 0.45 and 0.25, cell sizes between 200 and 250 nm and cell densities higher than 10^14^ cells/cm^3^. To produce these nanocellular polymers, the foaming parameters of the gas dissolution foaming technique using CO_2_ as blowing agent have been optimized. Taking into account previous works, the amount of CO_2_ uptake was maintained constant (31% by weight) for all the materials. Foaming parameters were modified between 40 °C and 110 °C for the foaming temperature and from 1 to 5 min for the foaming time. Foaming temperatures in the range of 80 to 100 °C and foaming times of 2 min allow for production of nanocellular polymers with relative densities as low as 0.25. Cellular structure has been studied in-depth to obtain the processing-cellular structure relationship. In addition, it has been proved that the glass transition temperature depends on the cellular structure. This effect is associated with a confinement of the polymer in the cell walls, and is one of the key reasons for the improved properties of nanocellular polymers.

## 1. Introduction

The research on cellular polymers is a popular topic in material science since the development of microcellular polymers in the 1980s at Massachusetts Institute of Technology (MIT) [1]. Nowadays, microcellular polymers (with cell sizes in the range of a few microns and cell densities around 10^9^ cells/cm^3^) are well known multiphasic materials. There are papers on the fabrication and characterization of different systems: polysulfone (PSU) [2], polystyrene (PS) [3], polyvinyl (chloride) (PVC) [4], polyurethane (PU) [5], polyethylene (PE) [6], polymethylmethacrylate (PMMA) [7], and polycarbonate (PC) [8]. The key reason that explains the interest in microcellular materials is that these materials improve the mechanical properties of conventional cellular polymers. This has been reported by different authors for different systems such as poly (ethylene terephthalate) (PET) [9], acrylonitrile butadiene styrene (ABS) [10], PVC [11] or PC [12]. In fact, these materials present better tensile and impact properties than conventional cellular polymers. To further improve the mechanical properties of these systems, there are two promising strategies: (1) improving the cellular structure by means of increasing the homogeneity of the cellular structure; and (2) reducing the average cell size [13,14]. This is one of the reasons that explains the significant interest that has appeared in the last few years in the development of nanocellular polymers. Nanocellular polymers are characterized by cell sizes below 300 nm and cell densities higher than 10^14^ cells/cm^3^ [15,16]. It is expected that a reduction in cell size to the nanoscale will provide materials with superior properties. In fact, the high potential of these materials has been recently reported for different systems. Miller et al. have proven that cell size reduction to the nanoscale in polyetherimide (PEI) results in an increase in strain to failure and tensile toughness [17]. In addition, it has been proved that nanocellular PMMA presents higher modulus of elasticity, higher impact strength and improved hardness than microcellular PMMA [18]. In addition, another interesting fact recently proved for nanocellular PMMA is that cell size reduction allows for decreasing the thermal conductivity due to the Knudsen effect [19,20].

Mechanical properties of cellular polymers depend on the cellular structure but relative density also plays an important role [21]. Therefore, it is essential to design production methods able to control both the cellular structure (generating cells with sizes in the nanoscale) as well as the density.

Several methods have been proposed to produce nanocellular polymers. For instance, pattern-transfer techniques have been used to obtain thin film nanocellular polymeric materials [22]. Another approach is the use of solvent based techniques in which nanocellular polymers are fabricated from block copolymers with thermally stable blocks and thermally labile blocks. The thermally labile blocks are removed by using organic solvents leaving nanopores behind [23]. This route works with high *T*_g_ polymers/copolymers systems. However, one of the most promising techniques in the production of bulk nanocellular polymers is the gas dissolution foaming process, usually using CO_2_ as blowing agent. This technique involves the saturation of the polymer by the gas phase in high pressure atmospheres and the release of the pressure when the polymer is saturated. When the polymer is under atmospheric pressure again, its super saturation state results in a nucleation process. The nucleation sites are able to grow, typically by heating the polymer over its effective glass transition temperature [24]. There are several studies that have used this technique to create nanocellular polymers. Nanocellular materials produced from pure polymers can be found in systems such as PEI, studied by Sundarram and Li [25]. They were able to produce nanocellular PEI with relative densities around 0.3 and 200 nm of cell size, using 8 MPa and 35 °C as saturation parameters. Nanocellular PMMA with 120 nm of cell size and a relative density of 0.23 has been fabricated by Guo et al. using a saturation process carried out at low temperatures (−20 °C) [22]. The same method has been also used by Guo et al. for polycarbonate, achieving 200 nm of cell size and 0.38 of relative density [26]. Another approach to produce nanocellular polymers using the gas dissolution technique is the use of nano-structured polymers as precursors for the foaming process. These materials have shown, to date, to be more appropriate for obtaining high cell densities using low saturation pressures and high saturation temperatures, but, on rare occasions, they present low relative densities. For instance, nanocellular PMMA/MAM (triblock copolymers poly(methyl methacrylate)-poly(butyl acrylate)-poly(methyl methacrylate)) blends produced by Pinto et al. had relative densities of 0.41 and a cell sizes around 200 nm [27]. They used 30 MPa and 25 °C as saturation conditions. Another PMMA copolymer, PMMA-oEA/SAN (styrene-acrylonitrile copolymer), studied by Costeux et al. was able to produce nanocellular polymers with relative densities of 0.4 and cell sizes of 100 nm. They used saturation conditions of 33 MPa and 30 °C [28]. Another strategy has been the introduction of nanoparticles as nucleating sites. Nanocellular polymers from PC with silica nanoparticles were fabricated by Zhai achieving cells with average sizes of 400 nm and 0.8 of relative density [29]. In addition, by using simultaneously homogeneous and heterogeneous nucleation mechanisms in PMMA-*co*-EMA containing nanoparticles, it has been possible to reduce relative density up to values of 0.2 obtaining cell sizes of 80 nm using 30 MPa as saturation pressure [30].

As it was described in the previous paragraphs, several types of nanocellular polymers have been fabricated up to now by using different systems. However, low relative densities are hardly found and, in the cases they were obtained, complex polymeric matrices such copolymers containing nanoparticles or they have used non-conventional processing parameters such as low saturation temperatures (i.e., saturation temperatures clearly below room temperature) have been used. In addition, in the previous published papers, the process-density-cellular structure relationship has not been analyzed in detail. In particular, the effect of reducing the density on structural characteristics such as cell size, cell nucleation density, cell size distribution, anisotropy ratio, fraction of mass in the struts or open cell content have not been described in detail in previous publications.

Taking the previous information into account, this paper has two main goals. The first one is to obtain nanocellular polymers with low relative density using a conventional PMMA homopolymer and saturation conditions that do not require low temperatures. The second one is to analyze in detail the process-density-cellular structure relationships for these novel materials.

## 2. Materials and Methods

### 2.1. Materials

Polymethylmethacrylate (PMMA) V 825T was kindly supplied by ALTUGLAS^®^ International (Colombes, France) in the form of pellets. The material used presents a density (ρ) of 1.19 g/cm^3^ (measured at 23 °C and 50% HR) and a glass transition temperature (*T*_g_) of 114.5 °C measured by DSC. Medical grade CO_2_ (99.9% purity) was used as blowing agent.

### 2.2. Methods

#### 2.2.1. Precursor Production

The as received PMMA was processed into sheets of (155 × 75 × 4 mm^3^) using a hot plate press. The process consists of three stages. The pellets were first heated at 250 °C during 9 min in the hot plates without applying any pressure. Then, they were pressed under a constant pressure of 2.2 MPa for another minute. Finally, the sheet was cooled down at room temperature under the same pressure.

These sheets were cut into 20 × 10 × 4 mm^3^ samples that were used as precursors for the foaming experiments.

#### 2.2.2. Foaming Tests

Foaming experiments were performed in a high pressure vessel (model PARR 4681) provided by Parr Instrument Company (Moline, IL, USA). The system to supply the gas pressure comprises an accurate pressure pump controller (model SFT-10) provided by Supercritical Fluid Technologies Inc (Newark, DE, USA). Thermal baths (J.P. Selecta Model 6000685, Grupo Selecta, Bercelona, Spain) have been used to heat the samples after saturation with CO_2_. A set of foaming experiments have been performed with this set-up using the so-called gas dissolution foaming process [24]. This production route consists of three stages, the saturation step, the desorption step and the foaming step. Samples are introduced in the pressure vessel under a high pressure atmosphere up to saturation. Then, the pressure is released and after some time (desorption time) samples are immersed in a thermal bath for foaming.

Saturation parameters have been chosen to achieve a 31% of CO_2_ uptake, an amount suitable to produce nanocellular materials in PMMA [18,31]. Saturation pressure (*p*_sat_) was fixed at 31 MPa and saturation temperature (*T*_sat_) at 25 °C. Saturation time was 24 h for all the experiments. After saturation, the pressure was released by using a fast depressurization rate (100 MPa/s), achieved by using an electrovalve with *K*_v_ = 1.1 L/min. Desorption time for all the experiments was 3 min. Foaming temperatures were modified from 40 °C to 110 °C and foaming times from 1 min to 5 min in order to study the influence of these parameters in the density and cellular structure.

#### 2.2.3. Amount of Gas Uptake

Gas uptake was calculated as the percentage of weight increment of the sample due to the gas sorption. The final weight of the samples after the whole saturation process was evaluated from the desorption vs. time curve, which was registered with a Mettler-Toledo balance (Mettler-Toledo, Columbus, OH, USA). This curve can be extrapolated to zero desorption time in order to obtain the total amount of gas uptake during saturation [32]. As it has been previously mentioned, the gas uptake for all the experiments was 31 ± 0.3% by weight.

#### 2.2.4. Density

Density of solid samples (ρ_s_) was measured with a gas pycnometer (Mod. AccuPyc II 1340, Micromeritics, Norcross, GA, USA), and density of cellular samples (ρ_f_) was determined using the water-displacement method based on Archimedes’ principle. A density determination kit for an AT261 Mettler-Toledo balance has been used for this purpose. Relative density (ρ_r_) has been calculated as ρ_f_/ρ_s_. The solid skin of nanocellular samples (that present maximum values of 100 µm) has been removed with a polisher (model LaboPOl2-LaboForce3, Struers (Ballerup, Dinamarca), by removing 200 µm on each side. This polishing process was performed before measuring the material density ρ_f_.

#### 2.2.5. Open Cell Content

The percentage of open cells (OC %) was measured with a gas pycnometer (Mod. AccuPyc II 1340, Micromeritics), according to ASTM D6226-10. The equation to calculate the open cell content is:

(1) $$O_{\nu }\left(\%\right)=\frac{V-V_{p}-V_{s}}{V\left(1-\rho_{r}\right)},$$

where *V* is the geometric volume of the sample, *V*_p_ is the volume measured by the pycnometer and *V*_s_ takes into account the exposed cells at the surface of the sample. The external volume, *V*, was determined from the cellular material density (and its mass (m) (measured with an AT261 Mettler-Toledo balance) as V=m/ρ. In order to determine *V*_p_, a pressure scan (from 0.2 MPa to 1.3 MPa) with the gas pycnometer has been performed measuring the pycnometric volume for each pressure. From a certain pressure, the volume remains constant, which demonstrates that no more gas can enter inside the cellular material. *V*_p_ has been considered as the mean value of these last constant values measured.

As *V*_s_ is proportional to the cell size, this value becomes negligible for nanocellular materials and can be neglected in these measurements.

#### 2.2.6. Scanning Electron Microscopy

To prepare the samples for SEM visualization, they were cooled down with liquid nitrogen and then fractured. In addition, they were coated with gold using a sputter coater (model SDC 005, Balzers Union, Balzers, Liechtenstein). An ESEM Scanning Electron Microscope (QUANTA 200 FEG, Hillsboro, OR, USA) has been used to obtain images of the cellular structure. The homogeneity of the cellular structure of the samples was analysed by taking different micrographs through the thickness. It was observed that samples were very homogeneous once the external solid skin was removed.

Cellular structure of each material was characterised with a software based on ImageJ/FIJI [33]. Structural parameters such as cell nucleation density (*N*_0_), calculated using the Kumar’s method, average cell size(ϕ), cell size distribution, standard deviation of the cell size distribution (SD), and anisotropy ratio (AR) calculated as the ratio of the cell size in the compression direction during precursors production (set as *z*-axis) and the cell size in a direction perpendicular to it (*x* direction) have been obtained [34]. A total of two micrographs randomly obtained have been used for the analysis of each material. Therefore, more than 300 cells have been considered for each specimen.

**Figure 1:**
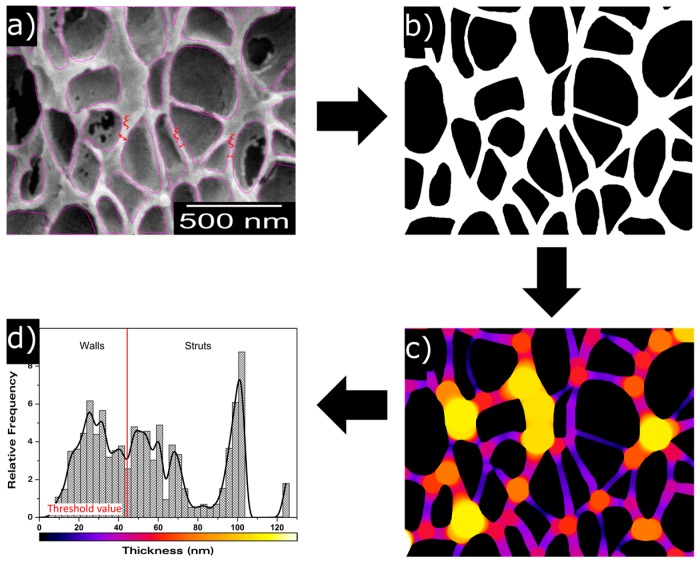
Description of the method to measure the fraction of mass in the struts (*f*_s_). (**a**) cell mask; (**b**) binarized cell mask; (**c**) local thickness cell image; (**d**) local thickness histogram.

With the aim of obtaining a more complete description of the cellular structure, some advanced cellular structure descriptors such as mean cell wall thickness (ξ) and mass fraction in the struts (f_s_) have been measured. Mean cell wall thickness has been measured directly from the micrographs. More than sixty cell walls have been measured per type of material. The average value has been used to characterize the material. This is a 2D characterization method, so broken walls cannot be easily detected in micrographs, thus the measured mean cell wall thickness values correspond to visible (non-broken) cell walls. *f*_s_ has been calculated using ImageJ/FIJI and the method explained in Figure 1. First of all, a representative region of the micrograph was cropped, then cells were marked in order to create a mask (Figure 1a). The second step consists of binarizing the created mask to isolate the solid phase and the gaseous one, marking the first one in white and the second one in black (Figure 1b). Local thickness can be analysed from this image, obtaining a local thickness image of the original cellular material (Figure 1c). In this image, due to the difference in thickness that struts and walls present, it is possible to distinguish these two different structural elements and to obtain a local thickness histogram (Figure 1d). This histogram quantifies the relative frequency corresponding to each thickness. In order to evaluate the fraction of material in the struts, a threshold value has been chosen as the minimum thickness corresponding to a strut (Figure 1d).

This minimum thickness was chosen by measuring the thickness of different struts directly from the micrographs and selecting the minimum value as threshold. For each image, it was confirmed that this threshold value was selected properly to avoid an overlapping of the two distributions, i.e., to avoid computing cell walls as struts. Finally, the fraction of mass in the struts can be determined as the total sum of relative densities corresponding to struts thickness. Some samples (those with lower densities) did not present sufficient differences in thickness between struts and cell walls (the two distributions showed a significant overlap), so for these particular samples, it was not possible to apply this quantification approach.

#### 2.2.7. Differential Scanning Calorimetry

Glass transition temperature (*T*_g_) has been measured by using a Mettler DSC30 differential-scanning calorimeter (Mettler-Toledo, Columbus, OH, USA) previously calibrated with indium. The *T*_g_ was taken as the mid-point of the change in the DSC thermogram that characterizes this transition. The weights of the samples were approximately 5 mg. To study the glass transition temperature of the as processed samples a first heating step was performed between 20 °C and 160 °C at 10 °C/min. Later on, samples were maintained at 160 °C for 3 min to erase any thermal history, and then they were cooled from 160 °C to 20 °C at −10 °C/min. Finally, the initial cycle of heating from 20 °C to 160 °C at 10 °C/min was performed again to determine the glass transition temperature of samples with the same thermal history. These experiments were done on the cellular materials and on the solid sheets. *T*_g_ increment $\left(\Delta T_{g}\right)$, defined as the difference between the *T*_g_ of the cellular material in the first heating step and that of the solid material in the same heating step, was calculated for each material. The same calculation was performed for the second heating step defining $\;\Delta T_{g_{2}}$.

## 3. Results

### 3.1. Influence of the Foaming Temperature and Time

#### 3.1.1. Relative Density

**Figure 2:**
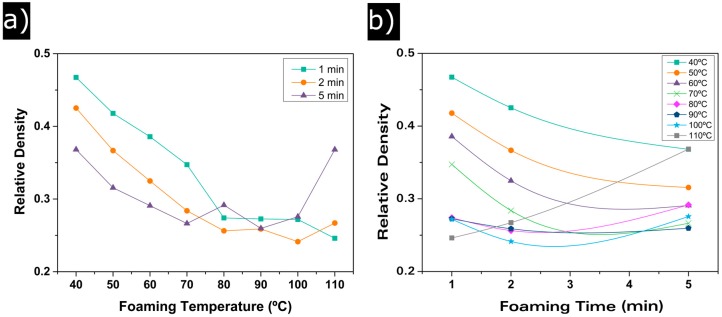
(**a**) relative density evolution with foaming temperature; (**b**) relative density evolution with foaming time.

To study the influence of the foaming parameters on the final cellular structure, different foaming temperatures have been used, from 40 °C to 110 °C, increasing in intervals of 10 °C. Furthermore, the influence of the foaming time has been determined using 1, 2 and 5 min of foaming time for each temperature. Relative density of each sample has been measured. As it can be seen in Figure 2, the relative density has a clear tendency with both the foaming temperature and the foaming time.

Relative density experiences an important decay from 0.46 at 40 °C of foaming temperature to a minimum of around 0.24 when the temperature increases. This decay seems to reach equilibrium at 80 °C. No significant differences in relative density are observed between samples foamed at 80 °C and the ones foamed at 90 °C and 100 °C. This is also true for samples foamed at 110 °C for foaming times of 1 and 2 min, but for 5 min relative density suffers a sharp increase (Figure 2a and Table 1).

Figure 2b shows the effect of foaming time. An increase in the foaming time results in a decrease in the relative density for temperatures from 40 °C to 80 °C. At this last temperature, the equilibrium reached by ρ_r_ is also detected. For higher temperatures, 90 °C and 100 °C, densities are similar for times between 1 and 5 min. It can be observed again that the experiments performed at 110 °C of foaming temperature do not follow the general trend; the lowest density is reached at 1 minute and then the relative density increases to values above 0.35.

It can be concluded that, for these saturation conditions, there exist a minimum relative density of 0.25 that can be reached using temperatures between 80 °C and 100 °C and foaming times between 2 and 5 min. Moreover, 110 °C seems to be the upper limit for the foaming temperature because at 5 min of foaming time, the relative density increases significantly. This is a consequence of the very close value of this temperature and the *T*_g_ of the used polymer.

#### 3.1.2. Cellular Structure

**Figure 3:**
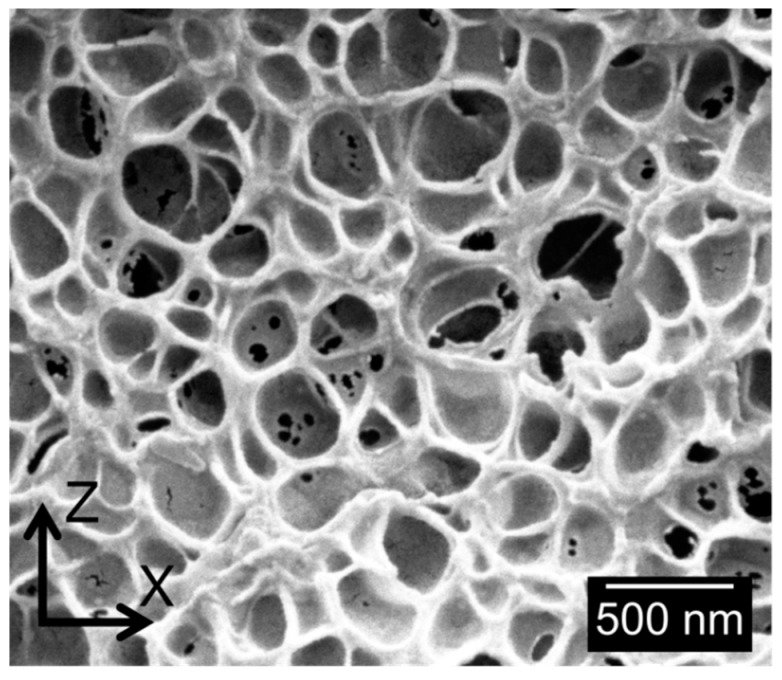
Micrograph of cellular structure of Sample 7.

One example of the typical cellular structures obtained is shown in Figure 3.

As it has been previously mentioned, samples present cell sizes in the nanometric range. The high homogeneity of the cellular structure, as well as a slight anisotropy of the cells in *z*-direction, can also be observed.

**Figure 4:**
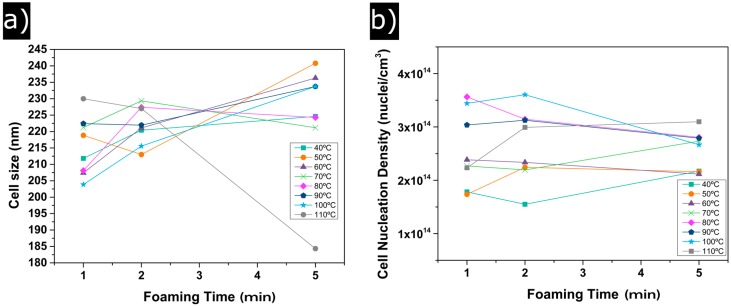
(**a**) evolution of the cell size with foaming time; (**b**) evolution of cell nucleation density with foaming time.

Figure 4 shows the evolution of the cell size (a) and cell nucleation density (b) with foaming time and foaming temperature. Between 40 °C and 100 °C of foaming time, cell sizes slightly increase from 205 to 240 nm (Figure 4a); consequently, only a small variation of 35 nm is detected. For a foaming temperature of 110 °C, only samples foamed during 1 and 2 min follow the general trend, whereas the cell size of the sample foamed during the 5 min experiment reduction due to the higher density of this material. It is also remarkable that the standard deviation of the cell size distribution divided by the average cell size (SD/Φ) (Table 1) remains constant for all temperatures and foaming times, with values near 0.4. Therefore, the homogeneity of the cellular structure does not depend on the foaming parameters, obtaining homogeneous cellular materials for all the foaming conditions.

Cell nucleation density tendencies are shown in Figure 4b. Two different behaviours can be distinguished. On the one hand, for foaming temperatures of 40 °C, 50 °C and 60 °C, the values of N_0_ are below 2 × 10^14^ cm^−3^. On the other hand, samples corresponding to foaming temperatures between 80 °C and 110 °C show higher values of *N*_0_ (above 3 × 10^14^ nuclei/cm^−3^). For instance, *N*_0_ increases from 1.5 × 10^14^ nuclei/cm^3^ for 40 °C of foaming temperature and 2 min of foaming time to 3.5 × 10^14^ nuclei/cm^−3^ for 100 °C of foaming temperature and the same foaming time.

Table 1 also shows the other characteristics measured: anisotropy ratio, cell wall thickness, mass fraction in the struts, open cell content and glass transition temperature increments. The anisotropy ratio (AR) is higher than one for all the materials under study. This indicates that cells are slightly elongated in the *z*-direction (i.e., the direction of the applied pressure during the production of the solid precursors). In addition, the obtained at temperatures below 60 °C are between 1.2 and 1.3, while for foaming temperatures higher than 60 °C AR are slightly reduced to values between 1.0 and 1.2. The values of cell wall thickness are in a range between 22 nm and 30 nm, except for sample 24 (the one foamed at high temperature and with high foaming times) that presents a higher value of 36nm. Therefore, ξ seems to remain almost constant with foaming temperature as well as with foaming time. In fact, there is no a clear trend of this parameter with foam density. Otherwise, mass fraction in the struts changes significantly with foaming temperature; the values are reduced from 0.6 to 0.3 when temperature is increased. As already discussed, this magnitude was not measured in some samples (low density samples) because of the similarities between the sizes of cell walls and struts.

#### 3.1.3. Open Cell Content

**Figure 5:**
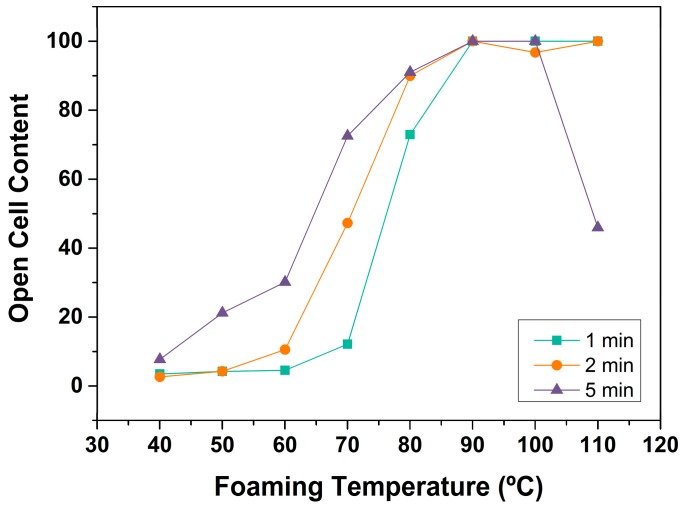
Open cell content as a function of foaming temperature.

The evolution of open cell content with foaming conditions is shown in Figure 5. Up to 80 °C of foaming temperature, OC increases from 3% at 40 °C to 91% at 80 °C. Temperatures of foaming higher than 80 °C yield to completely open cell structures. A tendency with the foaming time is also observed, and open cell content increases with this parameter. Cellular materials foamed during 5 min present higher open cell content than those foamed during 1 or 2 min. Once again, the nanocellular material produced at 110 °C of foaming temperature and 5 min of foaming time presents an anomalous behaviour. Due to the higher density and change in the internal cellular structure, the sample does not present a completely open cell structure.

#### 3.1.4. Glass Transition Temperature

Table 1 shows that when the glass transition temperature is measured in the first heating step, there are differences up to 11 °C between the glass transition of the nanocellular polymer and that of the solid precursor, while these differences disappear when the glass transition temperature is measured in the second heating step.

## 4. Discussion

As it has been described in the previous section, nanocellular polymers with a wide range of relative densities (from 0.47 to 0.24) have been produced. Consequently, the cellular structure of these cellular materials is different. In this section, the correlations found between density and the parameters that define the cellular structure are discussed. The section is divided into two parts—firstly the analysis of the characteristics related to the gaseous phase and secondly the study of the parameters connected to the solid phase.

### 4.1. Gaseous Phase

In order to analyze the relationship between relative density and the changes in gaseous phase, obtained results for the cell size and cell nucleation density should be discussed.

As mentioned above, the relative density changes in a factor of nearly 2, from 0.47 for the material with the highest density (sample 1) to 0.24 for the material with the lowest density (sample 20).

**Figure 6:**
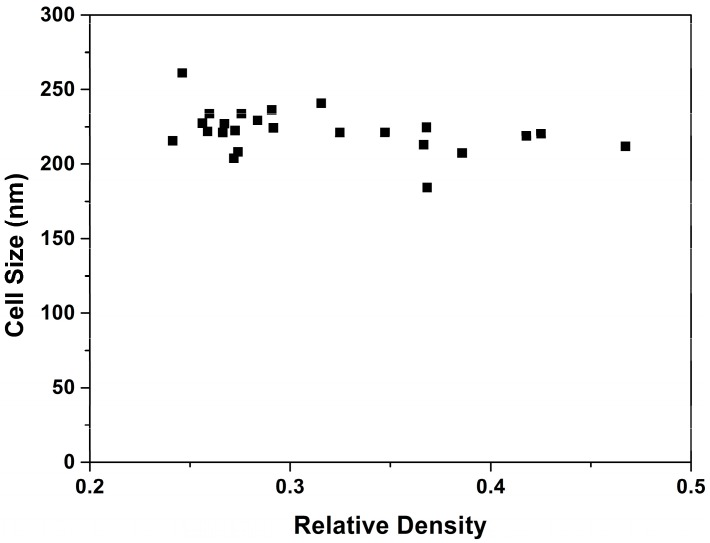
Average cell size as a function of relative density.

If the number of cells is constant, a change of a factor of two in relative density should be translated in a change of 1.26 (2^1/3^) in the cell size. However, the ratio of cell sizes between these samples (1 and 20) is only 1.02 (Table 1). This is just an example of the general trend observed (Figure 6).

Even though the range of relative densities is wide, almost all the samples have cell sizes in the same range.

**Figure 7:**
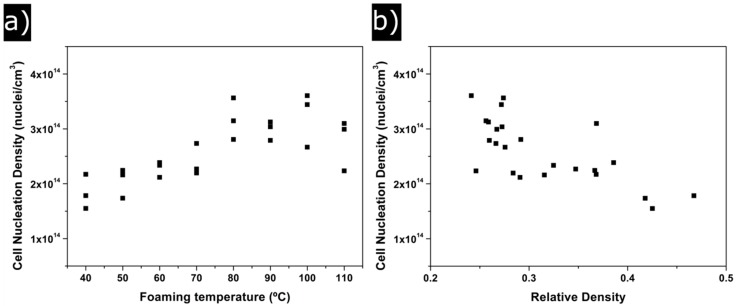
(**a**) change of cell nucleation density with foaming temperature; (**b**) change of cell nucleation density with relative density.

Cell nucleation density has also been analyzed. *N*_0_ changes from 1.5 × 10^14^ for samples produced at low temperatures to 3.5 × 10^14^ for samples produced at high temperatures, that is, *N*_0_ doubles its value when the foaming temperature is modified (Figure 7a).

Figure 7a shows N_0_ as a function of the foaming temperature. Cell nucleation density clearly increases between 40 °C and 80 °C, temperature at which the number of nucleation sites reaches its maximum value. Again, it is demonstrated that 110 °C is the upper limit for the foaming temperature because *N*_0_ drops again at this temperature. This is a behaviour opposite to that found for relative density (see Figure 2a). Between 40 °C and 80 °C, cell nucleation density grows due to a reduction in the energy barrier to create cells [8]. At higher temperatures, there is a competition between the higher nucleation rate and the emergence of degeneration mechanisms such as a coarsening, coalescence and/or collapse of the cellular structure. These last mechanisms seem to play a significant role when a temperature of 110 °C is used for foaming (cell nucleation density is reduced).

In short, the reduction in relative density is a result of an increase in the number of nucleation sites when the foaming temperature increases (Figure 7b). Cells created reach very similar sizes at any of the temperatures tested, so the increase in the volume of the samples produced at high temperatures is the result of having more cells (two times more cells) of equal size.

#### Open Cell Content

**Figure 8:**
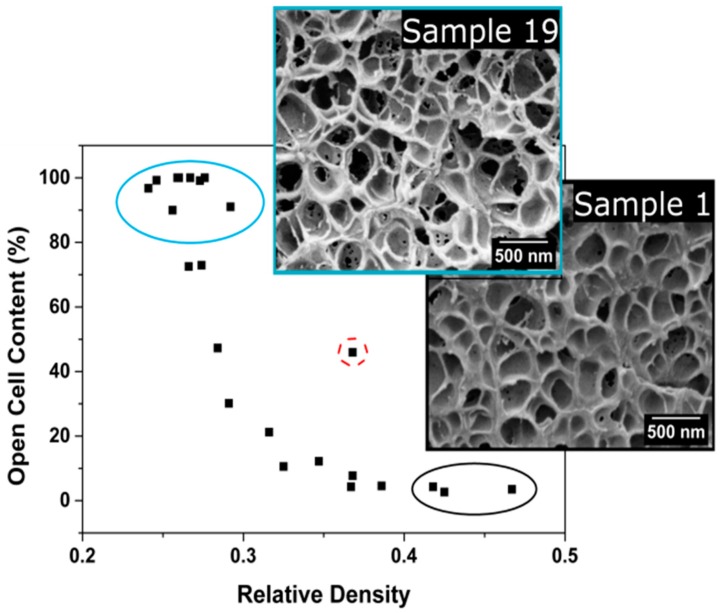
Open cell content as a function of relative density.

Figure 8 shows the relationship between the open cell content and the relative density. Samples with high relative densities present low contents of open cells. As density decreases, the open cell content increases up to a maximum value of 100%. Therefore, low density samples present a totally interconnected gas phase.

In previous sections, it has been proved that high temperatures are needed to decrease the relative density. These high temperatures imply a decrease in the polymer viscosity that causes cell wall ruptures during the expansion process.

Cell wall thickness has been measured to be constant, so it seems that there exists a minimum thickness value below which cell walls start to break down.

### 4.2. Solid Phase

**Figure 9:**
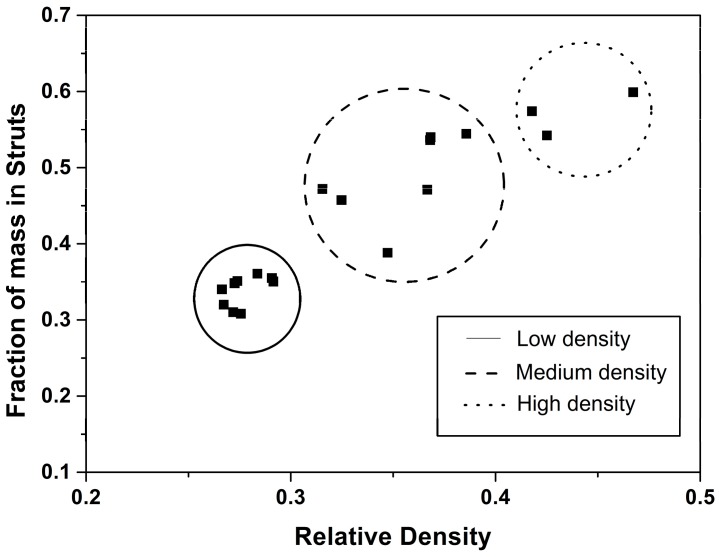
Fraction of mass in the struts with respect to relative density.

As a consequence of the evolution in the gaseous phase, the topology of the solid part of the cellular material is also modified. With the purpose of determining these modifications, parameters such as the cell wall thickness and the fraction of solid mass in the struts have been analysed. Mean cell wall thickness is almost constant for all the analysed materials (Table 1). In contrast, $f_{s}$ is strongly modified when density is reduced (Figure 9). When a cellular material reduces its relative density, increasing as a consequence its expansion ratio, it is common to expect a reduction in the cell wall thickness.

For example, in PU foams, it has been observed that a reduction in relative density implies drainage from the cell walls to the struts [35]. Then, cell walls become thinner as relative density decreases, reaching a minimum value at which coalescence starts to occur.

The results in Figure 9 and Table 1 demonstrate that a reduction in relative density results in totally different effects for nanocellular PMMA. As ρ_r_ decreases, the cell wall thickness remains constant, while the fraction of mass in the struts becomes smaller.

**Figure 10:**
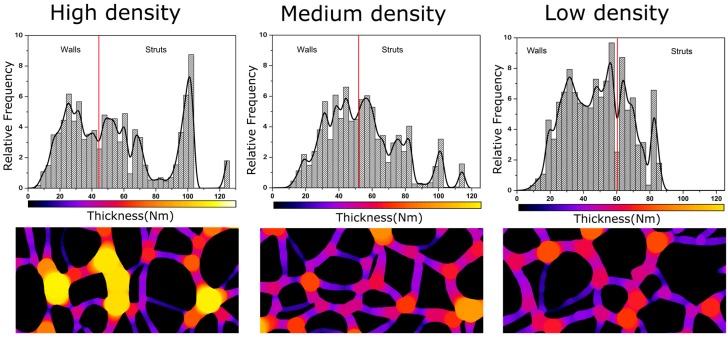
Solid phase distribution for high (Sample 1), medium (Sample 8) and low density samples (Sample 21).

As it is shown in Figure 9 and Figure 10, samples in this paper can be divided into three groups, taking into account their relative density and fraction of material in the struts: low density nanocellular materials, medium density nanocellular materials, and higher density ones. In order to clearly show the modifications in the local thickness, one example of the histograms of the local techiest for each group is included in Figure 10.

Modifications between different groups are clear. As it can be appreciated, mass fraction in the struts decreases as relative density falls. In fact, while in the high density materials, the struts have a clearly higher thickness, whereas in the low density nanocellular polymers, the struts have a thickness similar to that of the cell walls. In addition, the thickness distribution becomes narrow as the density is reduced. For low density materials, strut thickness is similar to cell wall thickness, which explains the fact that we were not able to accurately measure $f_{s}$ for the materials with the lowest densities.

Therefore, this analysis has proven that the key point in density reduction is to increase the foaming temperature, a parameter that induces a higher number of cells. This density reduction takes place by keeping constant the values of cell size and the cell wall thickness but reducing the fraction of material in the struts and increasing the interconnectivity of the cells.

#### Confinement Effect

As previously reported, there exist significant differences between the glass transition temperature of the nanocellular PMMA materials and that of the bulk material. This fact has been previously observed, and it is attributed to a confinement effect of the polymer matrix [18,36]. When the cellular polymers evolve from microcellular to nanocellular, higher cell densities, smaller cell sizes, and thinner cell walls, between 22 and 36 nm in our case, are obtained.

The size of these cell walls is of the same order of magnitude as the polymeric chain length, resulting in a confinement of the polymer within cell walls. This confinement restricts the mobility of the polymeric chains, making the *T*_g_ of the foamed material higher than the *T*_g_ of the solid precursor.

The importance of the confinement effect has recently been reported. It has been demonstrated that nanocellular materials present enhanced physical properties (modulus of elasticity, shore hardness), in comparison to microcellular ones, and this seems to be due, in part, to the confinement effect [18].

**Figure 11:**
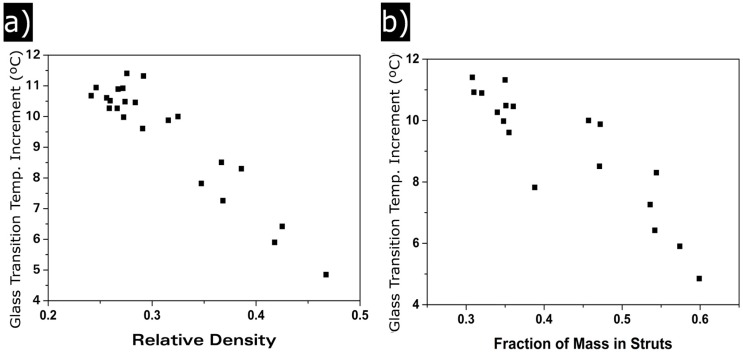
(**a**) glass transition temperature increment as a function of relative density; (**b**) glass transition temperature as a function of the fraction of mass in struts.

For the materials in this paper, c cell wall thickness remains constant independently of the relative density. Meanwhile, $\Delta T_{g}$ change in the studied range of relative densities (Figure 11). $\Delta T_{g}$ increases when the relative density is reduced, from 4 °C for the cellular materials with highest densities to 11 °C for cellular materials with the lowest densities.

Confinement effect is related to the solid phase of the cellular polymer, so although the cell wall thickness remains constant, it has been observed that *f*_s_ changes with $\rho_{r}$. Figure 11b shows that there is a clear relation between $\Delta T_{g}$ and the fraction of material in the struts. Reducing this value increases the $\Delta T_{g}$ values.

All the samples present confinement effect because thickness of the cell walls are of the same order of magnitude as that of PMMA chains. However, high density materials present a higher proportion of solid phase in the struts. In those areas, molecular mobility increases due to its higher size. As the struts size becomes smaller (i.e., density is reduced), the confinement starts to take part also in this part of the solid phase. This results in an increase of the confinement effect as relative density is reduced.

When a second measurement of the glass transition temperature is performed, after erasing the thermal history (Table 1), no differences between the nanocellular PMMA and the solid sheets are observed. This can be explained because when the nanocellular cellular structure disappears as a consequence of the increase in the temperature of the cellular material above its glass transition temperature (this was confirmed by performing SEM images of the samples after erasing the thermal history), the confinement effect disappears.

## 5. Conclusions

Low density nanocellular polymers have been fabricated using a PMMA homopolymer as raw material by means of the optimization of the foaming parameters. A wide range of relatives densities have been achieved, from 0.47 for 40 °C of foaming temperature to 0.24 for 90 °C of foaming temperature. A complete analysis of the cellular structure has been carried out, leading to a complete correlation process–density–structure. On the one hand, it has been found that cell sizes remain almost constant for all the samples, the increase of cell nucleation density being the key factor in the reduction of relative density. An increase in the foaming temperature from 40 °C to 90 °C increases the cell nucleation density by a factor of two, resulting in a reduction of the relative density of the same magnitude. In addition, it has been found that reducing the relative density increases the cells’ connectivity, and, in fact, for low relative density materials, the open cell content is 100%. On the other hand, whereas cell wall thickness is almost constant for all the produced material, maintaining a low value between 22 and 36 nm, the fraction of mass in the struts radically drops when the foaming temperature is increased and therefore when the relative density is reduced. Finally, it has been confirmed that the production of these nanocellular polymers with thin cell walls and struts results in a confinement effect of the polymeric matrix. The reduction of strut sizes when density is reduced causes a significant increase of this effect.

## Figures and Tables

**Table 1:** Production parameters and main characteristics of the materials under study.

Sample	F. Time (min)	F. Temp. (°C)	$\rho_{r}$	N_0_ (1/cm^3^)	ϕ (nm)	SD/ϕ	AR	ξ (nm)	*f*_s_	OC (%)	$\Delta T_{g}$ (°C)	$\Delta T_{g_{2}}$ (°C)
1	1	40	0.47	1.78 × 10^14^	212	0.45	1.24	26	0.60	4	4.9	0.1
2	2	40	0.43	1.55 × 10^14^	220	0.37	1.22	30	0.54	3	6.4	2.1
3	5	40	0.37	2.17 × 10^14^	225	0.39	1.26	24	0.54	8	7.3	0.7
4	1	50	0.42	1.74 × 10^14^	219	0.40	1.30	24	0.57	4	5.9	0.5
5	2	50	0.37	2.24 × 10^14^	213	0.41	1.32	26	0.47	4	8.5	0.8
6	5	50	0.32	2.16 × 10^14^	241	0.41	1.27	24	0.54	21	9.9	0.3
7	1	60	0.39	2.38 × 10^14^	207	0.42	1.21	26	0.46	5	8.3	1.6
8	2	60	0.33	2.34 × 10^14^	221	0.40	1.27	26	0.36	11	10.0	−0.7
9	5	60	0.29	2.12 × 10^14^	236	0.47	1.26	23	0.38	30	9.6	1.4
10	1	70	0.35	2.27 × 10^14^	221	0.44	1.24	24	0.39	12	7.8	1.2
11	2	70	0.28	2.19 × 10^14^	229	0.46	1.20	30	0.36	47	10.5	1.5
12	5	70	0.27	2.73 × 10^14^	221	0.44	1.28	25	0.34	73	10.3	0.3
13	1	80	0.27	3.56 × 10^14^	208	0.45	1.21	23	0.35	73	10.5	0.7
14	2	80	0.26	3.15 × 10^14^	227	0.40	1.14	26		90	11.6	2.2
15	5	80	0.29	2.81 × 10^14^	224	0.41	1.23	29	0.35	91	11.3	2.4
16	1	90	0.27	3.04 × 10^14^	222	0.40	1.12	29	0.35	99	10.0	0.5
17	2	90	0.26	3.13 × 10^14^	222	0.44	1.14	26		100	10.3	1.0
18	5	90	0.26	2.79 × 10^14^	234	0.40	1.09	28		100	10.5	0.6
19	1	100	0.27	3.44 × 10^14^	204	0.41	1.18	28	0.31	100	10.9	21.9
20	2	100	0.24	3.60 × 10^14^	216	0.46	1.03	26		97	10.7	2.5
21	5	100	0.28	2.67 × 10^14^	234	0.43	1.16	29	0.308	100	11.4	1.1
22	1	110	0.25	2.23 × 10^14^	261	0.43	1.19	28		99	10.9	1.4
23	2	110	0.27	2.99 × 10^14^	227	0.43	1.04	33	0.32	100	10.9	1.0
24	5	110	0.37	3.10 × 10^14^	184	0.40	1.17	36	0.54	46	4.9	2.0

## References

[1] Eaves D. (2004). Handbook of Polymer Foams.

[2] Sun H., Mark J.E. (2002). Preparation, characterization, and mechanical properties of some microcellular polysulfone foams. J. Appl. Polym. Sci..

[3] Arora K.A., Lesser A.J., McCarthy T.J. (1998). Preparation and characterization of microcellular polystyrene foams processed in supercritical carbon dioxide. Macromolecules.

[4] Kumar V., Weller J.E. (1993). A process to produce microcellular PVC. Int. Polym. Process..

[5] Parks K.L., Beckman E.J. (1996). Genetration of microcellular polyurethane foams via polymerization in carbon dioxide. II: Foam formation and characterization. Polym. Eng. Sci..

[6] Xing Z., Wu G., Huang S., Chen S., Zeng H. (2008). Preparation of microcellular cross-linked polyethylene foams by a radiation and supercritical carbon dioxide approach. J. Supercrit. Fluids.

[7] Reglero Ruiz J.A., Viot P., Dumon M. (2010). Microcellular foaming of polymethylmethacrylate in a batch supercritical CO_2_ process: Effect of microstructure on compression behavior. J. Appl. Polym. Sci..

[8] Kumar V., Weller J. (1994). Production of microcellular polycarbonate using carbon dioxide for bubble nucleation. J. Eng. Ind..

[9] Shimbo M., Higashitani I., Miyano Y. (2007). Mechanism of strength improvement of foamed plastics having fine cell. J. Cell. Plast..

[10] Nadella K., Kumar V. (2007). Tensile and flexural properties of solid-state microcellular ABS panels. Exp. Anal. Nano Eng..

[11] Juntunen R.P., Kumar V., Weller J.E., Bezubic W.P. (2000). Impact strength of high density microcellular poly(vinyl chloride) foams. J. Vinyl. Addit. Technol..

[12] Collias D.I., Baird D.G., Borggreve R.J. (1994). Impact toughening of polycarbonate by microcellular foaming. Polymer.

[13] Bureau M.N. (2006). Fracture toughness of high density polycarbonate microcellular foams. J. Cell. Plast..

[14] Kumar V., VanderWel M., Weller J., Seeler K.A. (1994). Experimental characterization of the tensile behavior of microcellular polycarbonate foams. J. Eng. Mater. Technol..

[15] Notario B., Pinto J., Rodriguez-Perez M.A. (2016). Nanoporous polymeric materials: A new class of materials with enhanced properties. Prog. Mater. Sci..

[16] Costeux S. (2014). CO_2_-blown nanocellular foams. J. Appl. Polym. Sci..

[17] Miller D., Kumar V. (2011). Microcellular and nanocellular solid-state polyetherimide (PEI) foams using sub-critical carbon dioxide II. Tensile and impact properties. Polymer.

[18] Notario B., Pinto J., Rodriguez-Perez M.A. (2015). Towards a new generation of polymeric foams: PMMA nanocellular foams with enhanced physical properties. Polymer.

[19] Schmidt D., Raman V.I., Egger C., du Fresne C., Schädler V. (2007). Templated cross-linking reactions for designing nanoporous materials. Mater. Sci. Eng. C.

[20] Notario B., Pinto J., Solorzano E., De Saja J.A., Dumon M., Rodriguez-Perez M.A. (2015). Experimental validation of the Knudsen effect in nanocellular polymeric foams. Polymer.

[21] Gibson L.J., Ashby M.F. (1997). Cellular Solids: Structure and Properties.

[22] Guo H., Nicolae A., Kumar V. (2015). Solid-state poly(methyl methacrylate) (PMMA) nanofoams. Part II: Low-temperature solid-state process space using CO_2_ and the resulting morphologies. Polymer.

[23] Hedrick J.L., Carter K.R., Cha H.J., Hawker C.J., DiPietro R.A., Labadie J.W., Miller R.D., Russell T.P., Sanchez M.I., Volksen W. (1996). High-temperature polyimide nanofoams for microelectronic applications. React. Funct. Polym..

[24] Martini-Vvedensky J.J.E., Suh N.N.P., Waldman F.F.A. (1984). Microcellular closed cell foams and their method of manufacture. US Patent.

[25] Zhou C., Vaccaro N., Sundarram S.S., Li W. (2012). Fabrication and characterization of polyetherimide nanofoams using supercritical CO_2_. J. Cell. Plast..

[26] Guo H., Kumar V. (2015). Some thermodynamic and kinetic low-temperature properties of the PC-CO_2_ system and morphological characteristics of solid-state PC nanofoams produced with liquid CO_2_. Polymer.

[27] Pinto J., Dumon M., Pedros M., Reglero J., Rodriguez-Perez M.A. (2014). Nanocellular CO_2_ foaming of PMMA assisted by block copolymer nanostructuration. Chem. Eng. J..

[28] Costeux S., Khan I., Bunker S.P., Jeon H.K. (2014). Experimental study and modeling of nanofoams formation from single phase acrylic copolymers. J. Cell. Plast..

[29] Zhai W., Yu J., Wu L., Ma W., He J. (2006). Heterogeneous nucleation uniformizing cell size distribution in microcellular nanocomposites foams. Polymer.

[30] Costeux S., Jeon M.H., Bunker T.S., Khan I. Nanocellular foams from acrylic polymers: Experiments and modeling. Society of Plastics Engineers FOAMS.

[31] Guo H., Kumar V. (2015). Solid-state poly(methyl methacrylate) (PMMA) nanofoams. Part I: Low-temperature CO_2_ sorption, diffusion, and the depression in PMMA glass transition. Polymer.

[32] Tang M., Du T.-B., Chen Y.-P. (2004). Sorption and diffusion of supercritical carbon dioxide in polycarbonate. J. Supercrit. Fluids.

[33] Pinto J., Solorzano E., Rodriguez-Perez M.A., de Saja J.A. (2013). Characterization of the cellular structure based on user-interactive image analysis procedures. J. Cell. Plast..

[34] Kumar V., Suh N.P. (1990). A process for making microcellular thermoplastic parts. Polym. Eng. Sci..

[35] Pardo-Alonso S., Solórzano E., Brabant L., Vanderniepen P., Dierick M., Van Hoorebeke L., Rodríguez-Pérez M.A. (2013). 3D Analysis of the progressive modification of the cellular architecture in polyurethane nanocomposite foams via X-ray microtomography. Eur. Polym. J..

[36] Reglero Ruiz J.A., Dumon M., Pinto J., Rodriguez-Pérez M.A. (2011). Low-density nanocellular foams produced by high-pressure carbon dioxide. Macromol. Mater. Eng..

